# The importance of nanoscale confinement to electrocatalytic performance†

**DOI:** 10.1039/c9sc05611d

**Published:** 2019-12-11

**Authors:** Johanna Wordsworth, Tania M. Benedetti, Ali Alinezhad, Richard D. Tilley, Martin A. Edwards, Wolfgang Schuhmann, J. Justin Gooding

**Affiliations:** School of Chemistry, Australian Centre for NanoMedicine, University of New South Wales Sydney 2052 Australia justin.gooding@unsw.edu.au r.tilley@unsw.edu.au; Electron Microscope Unit, Mark Wainwright Analytical Centre, University of New South Wales Sydney 2052 Australia; Department of Chemistry, University of Utah Salt Lake City UT 84112 USA martin.edwards@utah.edu; Analytical Chemistry – Center for Electrochemical Sciences (CES), Faculty of Chemistry and Biochemistry, Ruhr University Bochum Universitätsstr. 150 D-44780 Bochum Germany wolfgang.schuhmann@ruhr-uni-bochum.de; Australian Research Council Centre of Excellence in Convergent Bio-Nano Science and Technology, University of New South Wales Sydney 2052 Australia

## Abstract

Electrocatalytic nanoparticles that mimic the three-dimensional geometric architecture of enzymes where the reaction occurs down a substrate channel isolated from bulk solution, referred to herein as nanozymes, were used to explore the impact of nano-confinement on electrocatalytic reactions. Surfactant covered Pt–Ni nanozyme nanoparticles, with Ni etched from the nanoparticles, possess a nanoscale channel in which the active sites for electrocatalysis of oxygen reduction are located. Different particle compositions and etching parameters allowed synthesis of nanoparticles with different average substrate channel diameters that have varying amounts of nano-confinement. The results showed that in the kinetically limited regime at low overpotentials, the smaller the substrate channels the higher the specific activity of the electrocatalyst. This is attributed to higher concentrations of protons, relative to bulk solution, required to balance the potential inside the nano-confined channel. However, at higher overpotentials where limitation by mass transport of oxygen becomes important, the nanozymes with larger substrate channels showed higher electrocatalytic activity. A reaction-diffusion model revealed that the higher electrocatalytic activity at low overpotentials with smaller substrate channels can be explained by the higher concentration of protons. The model suggests that the dominant mode of mass transport to achieve these high concentrations is by migration, exemplifying how nano-confinement can be used to enhance reaction rates. Experimental and theoretical data show that under mass transport limiting potentials, the nano-confinement has no effect and the reaction only occurs at the entrance of the substrate channel at the nanoparticle surface.

## Abstract 2

Nanoparticles mimicking the three-dimensional architecture of enzymes where the reaction occurs down a channel isolated from bulk solution, referred here as nanozymes, were used to explore the impact of nano-confinement on electrocatalytic reactions.

## Graphical abstract

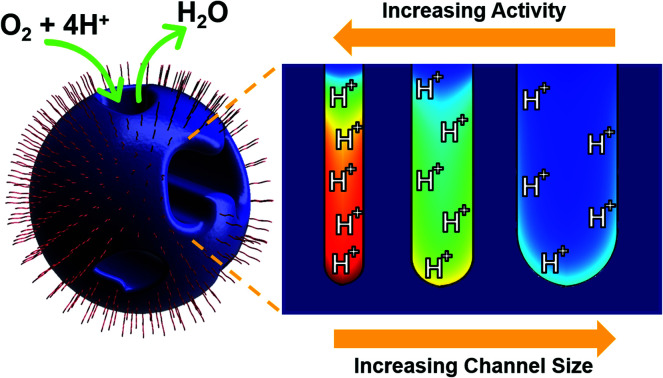

## Introduction

The analogies between enzymes and nanoparticles for catalysis are ever increasing with similar reactions being catalyzed and the physical size of these two families of catalysts also being similar.^1^ This has seen the rise of the term nanozymes, to represent nanoparticles that catalyze the same reactions as enzymes.^2,3^ Recently we extended the nanozyme concept to nanoparticles that mimicked the three-dimensional geometry of enzymes for electrocatalysis.^4^ This means that nanoparticles were synthesized with isolated substrate channels that penetrated into the center of the nanoparticle, with the exterior surface electrochemically passivated by a surfactant such that the electrochemical reaction occurs within a nano-confined substrate channel. Isolating the reaction center from the bulk solution provides a means to have control over the solution environment where the reaction proceeds.

In our initial study, we used platinum–nickel nanoparticles for the oxygen reduction reaction (ORR),^4^ due to their well-studied ORR catalysis^5^ and large lattice mismatch between the two metals, leading to nickel rich domains.^6^ Etching nickel in an acid gives isolated substrate channels with diameters of a couple of nanometers.^4^ Due to the use of oleylamine as the capping agent, the electrocatalytic reaction only proceeded within the substrate channels with the specific activity for the ORR being 3.3 times higher at 0.95 V (RHE) than when the surface outside the channels was exposed.^7–11^

The fact that mesoporous Pt–Ni nanoparticles, with a similar curvature, but interconnected pores, showed lower specific activity than the nanozymes with isolated substrate channels, suggested that nano-confinement and its impact on the solution conditions within the substrate channel are responsible for the observed increased electrocatalytic activity of these nanozymes. As such, the purpose of this paper was to explore the impact of nano-confinement within the substrate channels on the ORR activity of nanozymes. To achieve this, nanozymes with different average substrate channel diameters were synthesized and the impact of the channel dimensions on the ORR activity was evaluated. Physicochemical modelling shows similar trends to the experimental results and points to both reactant concentration inside and mass transport into the substrate channel being affected by the channel diameter. These results and understanding have significant implications for catalysts where nanoscale confinement can be used to increase catalytic activity.

## Results

**Fig. 1:**
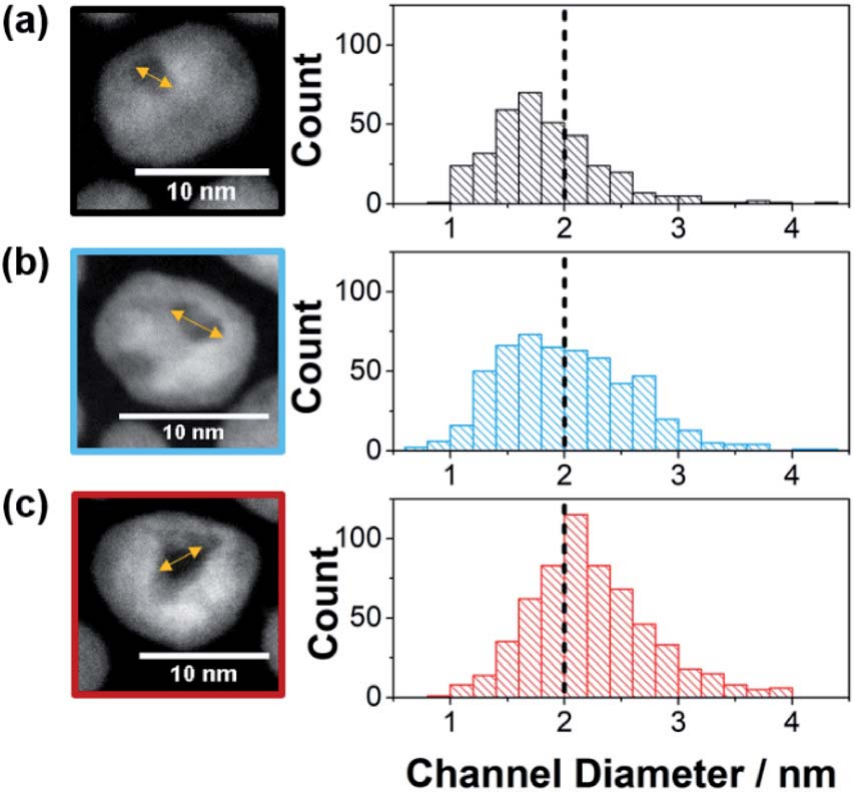
Channel diameter histograms with example HRTEM images used to measure the channel diameter for (a) NZ_small_, (b) NZ_medium_, and (c) NZ_large_. Note that for the three channel distributions, the percentage of channels below 2 nm in diameter were for NZ_small_ 68.5% (s.d. = 0.5 nm, *n* = 347), NZ_medium_ 51.7% (s.d. = 0.6 nm, *n* = 537) and NZ_large_ 33.9% (s.d. = 0.5 nm, *n* = 600). Further examples of HAADF_STEM images are found in Fig. S1.†

Pt–Ni nanozymes with three different distributions of substrate channel diameters were synthesized using a similar method to that described previously.^4,12^ Pt–Ni nanoparticles were synthesized in oleylamine which remained on the outer surface of the nanoparticles. The lower affinity of oleylamine for Ni rich domains was exploited to allow the less noble Ni to be etched out of the nanoparticles leaving a substrate channel with a platinum skin and the outside surface passivated.^4^ Nanozymes with different distributions of substrate channel diameters, as shown in Fig. 1, were obtained by altering the ratio of Pt to Ni used to synthesize the nanoparticles. By increasing the ratio of Pt : Ni from 1 : 1.5 to 1 : 2.5 and then to 1 : 3, an increase in the amount of Ni removed from the particles during the etching process was observed (31 wt%, 48 wt% and 65 wt% removed respectively as determined by ICP-OES). This resulted in a shift in the proportion of the substrate channel diameters that are below 2 nm as presented in the histograms in Fig. 1 (see ESI Fig. S1† for HAADF-STEM images of more nanozymes).

For the 1 : 1.5 ratio, the fraction of substrate channels that were below 2 nm was 69% whilst it was 52% and 34% for the 1 : 2.5 and 1 : 3 ratios, respectively. These will henceforth be referred to as NZ_small_, NZ_medium_ and NZ_large_ (Fig. 1). This Pt : Ni ratio range was chosen because ratios with more Pt resulted in no channels while higher Ni ratios resulted in mesoporous particles. The differences in the channel size distribution is also evident from the ratio between the ECSA measured from H_UPD_ and Cu_UPD_ (see ESI Fig. S2†). As the surfactant layer is partially permeable to H^+^ but blocks Cu^2+^, ECSA_HUPD_/ECSA_CuUPD_ gives an indication of the relative electrochemically available area of the channels with respect to the total nanozyme area. The value of ECSA_HUPD_/ECSA_CuUPD_ increases with increasing channel size distribution, being 0.12, 0.19 and 0.29 for NZ_small_, NZ_medium_ and NZ_large_, respectively. As a control to explore the effect of the nano-confinement in isolated substrate channels, mesoporous Pt–Ni nanoparticles, also with their exterior surface passivated, were obtained by changing the etching conditions (TEM in ESI Fig. S1d†).

**Fig. 2:**
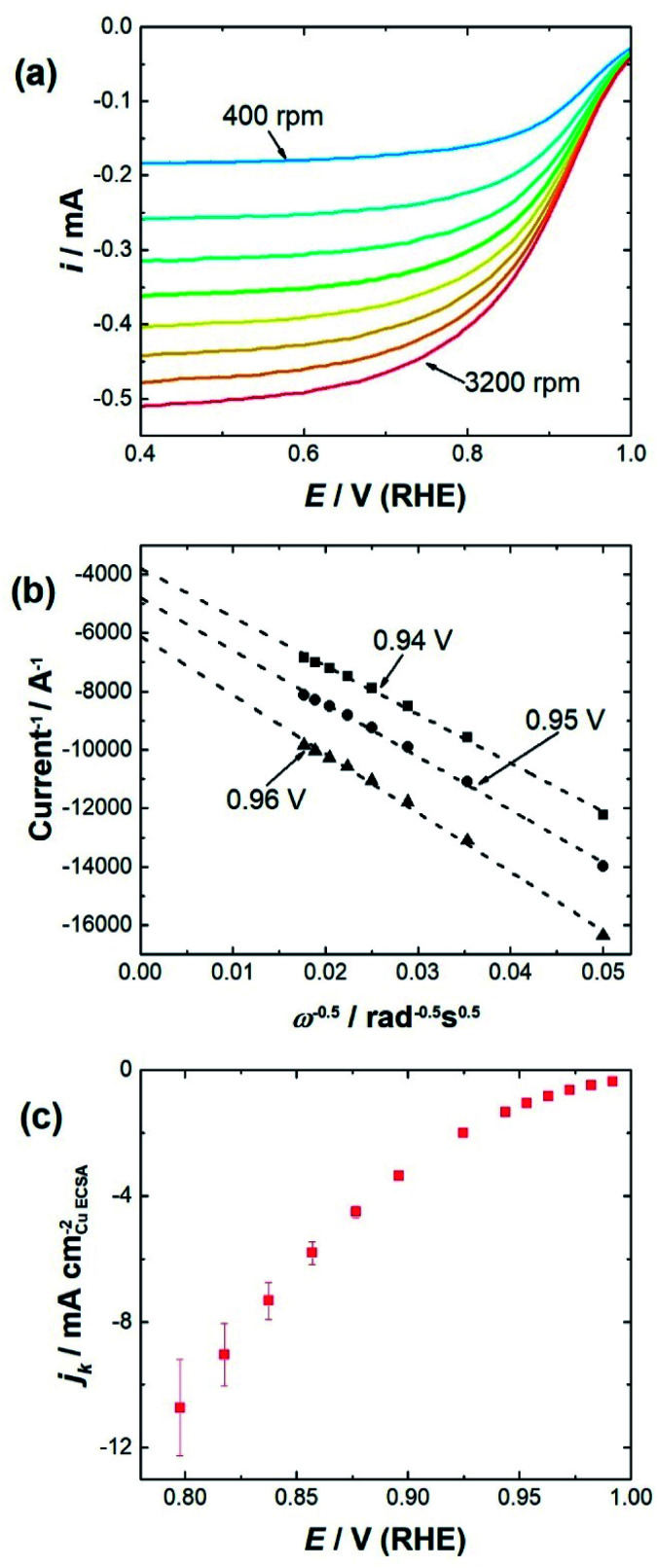
(a) Background subtracted and *iR* corrected LSVs of NZ_large_ at rotation speeds from 400–3200 rpm (400 rpm steps) in an O_2_ saturated electrolyte. The scans were performed in 0.1 mol L^−1^ HClO_4_ in the anodic direction at a scan rate of 100 mV s^−1^. (b) Example of a Koutecký–Levich plot for one measurement with NZ_large_ at 0.94 V, 0.95 V, and 0.96 V. Reciprocal of the intercept value is the kinetic current density at that potential, as the rotation speed is deemed to be infinite. (c) Kinetic current densities at different overpotentials for NZ_large_. The kinetic current densities are an average of measurements from three independently prepared electrodes.

To determine the impact of substrate channel diameter distribution on the electrocatalytic performance of the nanozymes, glassy carbon rotating disc electrodes were modified with carbon supported nanozymes and linear sweep voltammograms were recorded at different rotation speeds. TEM of the carbon supported nanozymes shows that their morphology is maintained and there is no agglomeration after the electrochemical measurements (Fig. S3†). The voltammograms of NZ_large_ in Fig. 2a show that the reaction reaches a mixed kinetic-diffusion controlled regime at relatively low overpotentials at around 0.9 V (RHE). The voltammograms of NZ_medium_ and NZ_small_ show similar behavior (ESI, Fig. S4†). In order to evaluate the specific activity of the reaction on the sites down the substrate channels, the kinetic currents were obtained by extrapolating the Koutecký–Levich plots obtained at different potentials to infinite rotation speeds,^13^ as shown in Fig. 2b at 0.96 V, 0.95 V and 0.94 V for NZ_large_ as an example. The kinetic current densities at potentials ranging from 0.8 V to 1.0 V were then plotted for the different nanozymes and the mesoporous particles (see Fig. 2c as an example for NZ_large_).

**Fig. 3:**
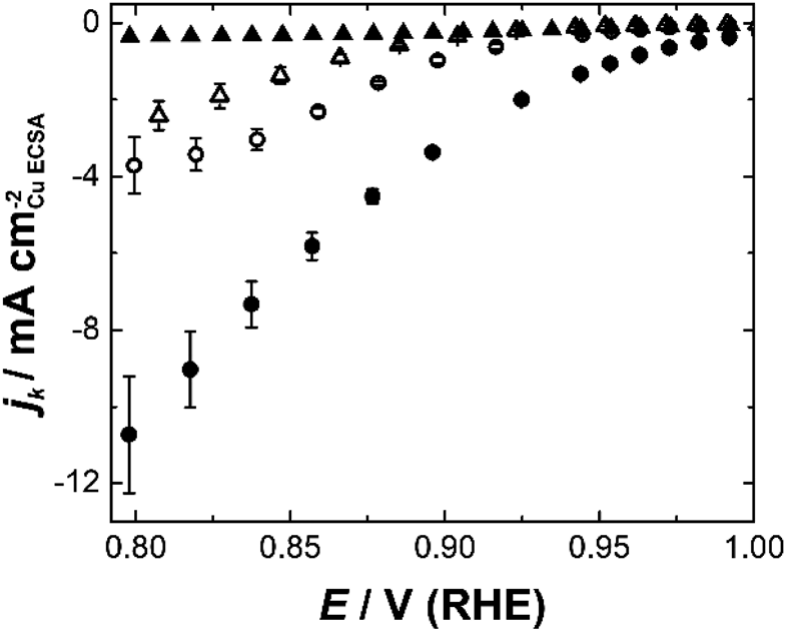
Kinetic current densities for NZ_large_ calculated using Koutecký–Levich plots from 0.8–1.0 V (RHE) for surfactant covered particles (filled shapes), particles with the surfactant removed (open shapes), etched particles with large channels – nanozymes (circles), and unetched particles (triangles).

Etched nanoparticles with the surfactant removed, non-etched particles with and without the surfactant and the mesoporous particles were also evaluated as controls for the nanozymes. Consistent with our previous study,^4^ the nanozymes show much higher specific activities than the other particles as seen in Fig. 3 for NZ_large_ (see ESI Fig. S5† for the equivalent data for the other NZs and the mesoporous particles). The non-etched nanoparticles with the surfactant are mostly inactive, confirming the ability of the surfactant to block the surface for the ORR. The fact that the etched particles with the surfactant removed are more active than the non-etched nanoparticles with the surfactant removed shows that the active sites inside the channels contribute to the overall activity over the entire potential range studied.

**Fig. 4:**
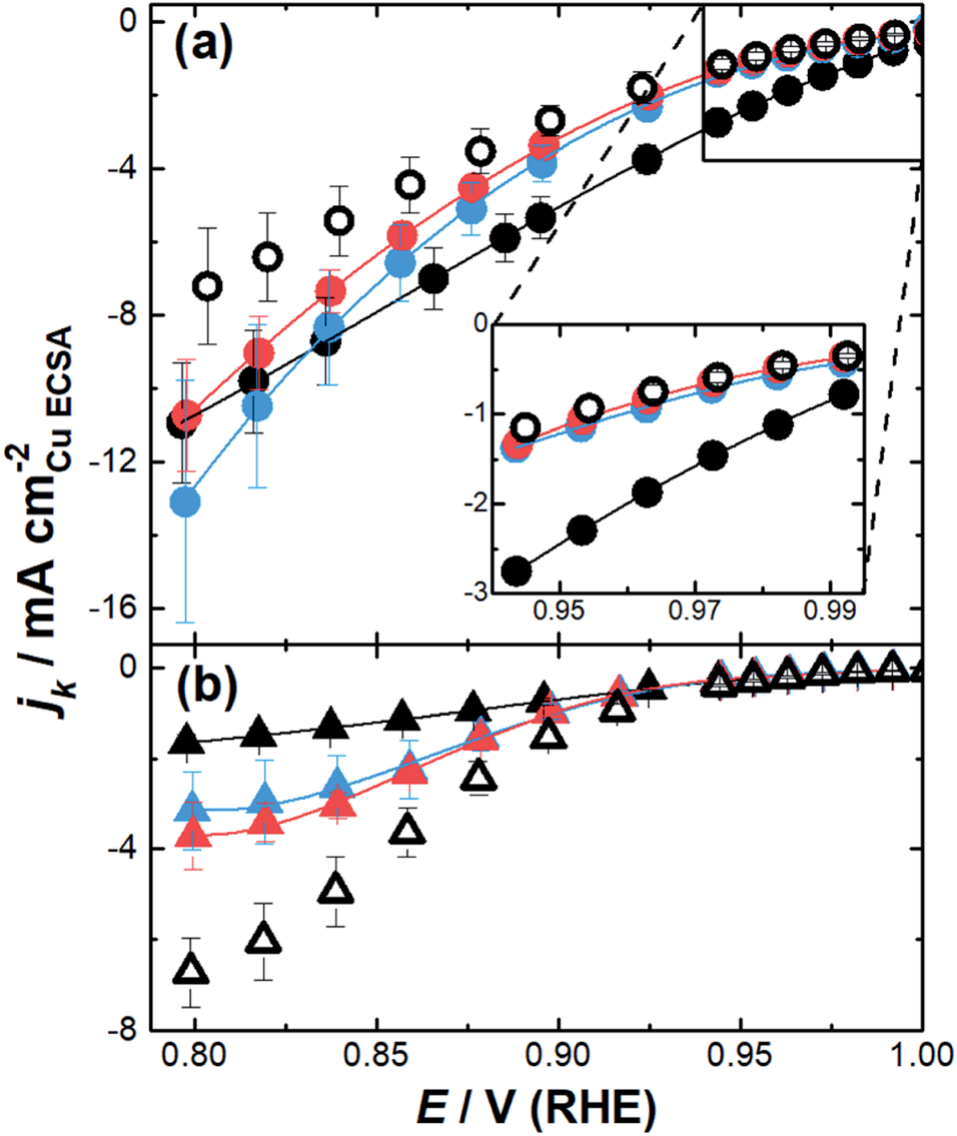
Kinetic current densities calculated from Koutecký–Levich plots from 0.8–1.0 V (RHE) for (a) NZ_small_ (black), NZ_medium_ (blue), NZ_large_ (red), and mesoporous particles (white); (b) small channel particles without the surfactant (black), medium channel particles without the surfactant (blue), large channel particles without the surfactant (red), and mesoporous particles without the surfactant (white). The error bars in the inset are smaller than the size of the dots.

The impact of the substrate channel diameter was then investigated for the nanozymes with three different channel diameter distributions (Fig. 4a). The results with mesoporous particles with the surfactant were also included for comparison to demonstrate the importance of isolated substrate channels over interconnected nanopores. Evidently, there are significant differences in the specific activity as a function of potential for the nanozymes with different average substrate channel diameters.

At low overpotentials, where electron transfer kinetics dominate (region in the inset of Fig. 4a), NZ_medium_ and NZ_large_ show very similar specific activity whilst NZ_small_ has significantly higher specific activity. In the mixed kinetic-diffusion potential regime where mass transport becomes significant, the reaction becomes progressively faster for NZ_medium_ and NZ_large_ with NZ_medium_ eventually becoming more active than NZ_small_.

These results show that different factors affect the ORR kinetics at low overpotentials and at higher overpotentials. While smaller channel diameters provide superior activity when the reaction is mostly dominated by the catalytic site's intrinsic activity, the nanozymes with larger channels are more active when the reaction starts to be significantly influenced by mass transport. The mesoporous particles with the outside surface coated with the surfactant present similar activity to the nanozymes with larger channels at low overpotentials but are significantly less active than all nanozymes at higher overpotentials.

When the surfactant was removed, the nanoparticles show quite different behavior (Fig. 4b). Firstly, the specific activity was lower than that of the nanozymes regardless of the channel dimensions in the mixed kinetic-diffusion potential regime. Secondly, at higher overpotentials the specific activity increases with the substrate channel diameter. Thirdly, the specific activity appears to reach a plateau for all etched nanoparticles without the surfactant which is not observed with the nanozymes. These observations will be explained in more detail below but relate to where the reaction proceeds on the nanoparticles relative to the actual surface area determined using Cu_UPD_. When the surfactant is removed the ORR can proceed on the outside of the nanoparticles but may not necessarily occur throughout the entire internal porous structure.

The results shown in Fig. 4 evoke some important questions. Firstly, why is the specific activity higher for NZ_small_ than the other nanozymes at low overpotentials? And why at higher overpotentials do the nanozymes with larger channels begin to show higher specific activity? In our previous study we hypothesized that the higher specific activity could be due to strain effects giving different active sites as a function of diameter or solution effects in the nano-confined channels.^4^ The lower specific activity with the mesoporous particles points towards a solution effect related to the nano-confinement within the isolated substrate channels of the nanozymes.

To elucidate whether the experimental observations of the overall higher specific activity of the nanozymes and the influence of the channel diameter when the reaction is under predominantly kinetic or mass transport control can be explained by nano-confinement, a physicochemical model was developed to investigate whether the experimental behavior can qualitatively be replicated by a simple model that considers the concentration of solution species, the geometric space and where the potential and the current response of a single channel within a nanozyme were calculated by solving the coupled Poisson–Nernst–Planck equations. The reaction scheme assumes a 4 electron reduction of oxygen rather than considering any 2 electron reduction to give hydrogen peroxide. This was chosen to keep the model as simple as possible but is justified because hydrogen peroxide production is expected when protons are limiting and, as seen below, this is not observed in the kinetic region of the voltammograms. Small and large fixed charges were applied to mimic the reaction happening in predominant kinetic and mass transport regimes, respectively (see the ESI and Fig. S6 and S7† for a detailed description of the model).

**Fig. 5:**
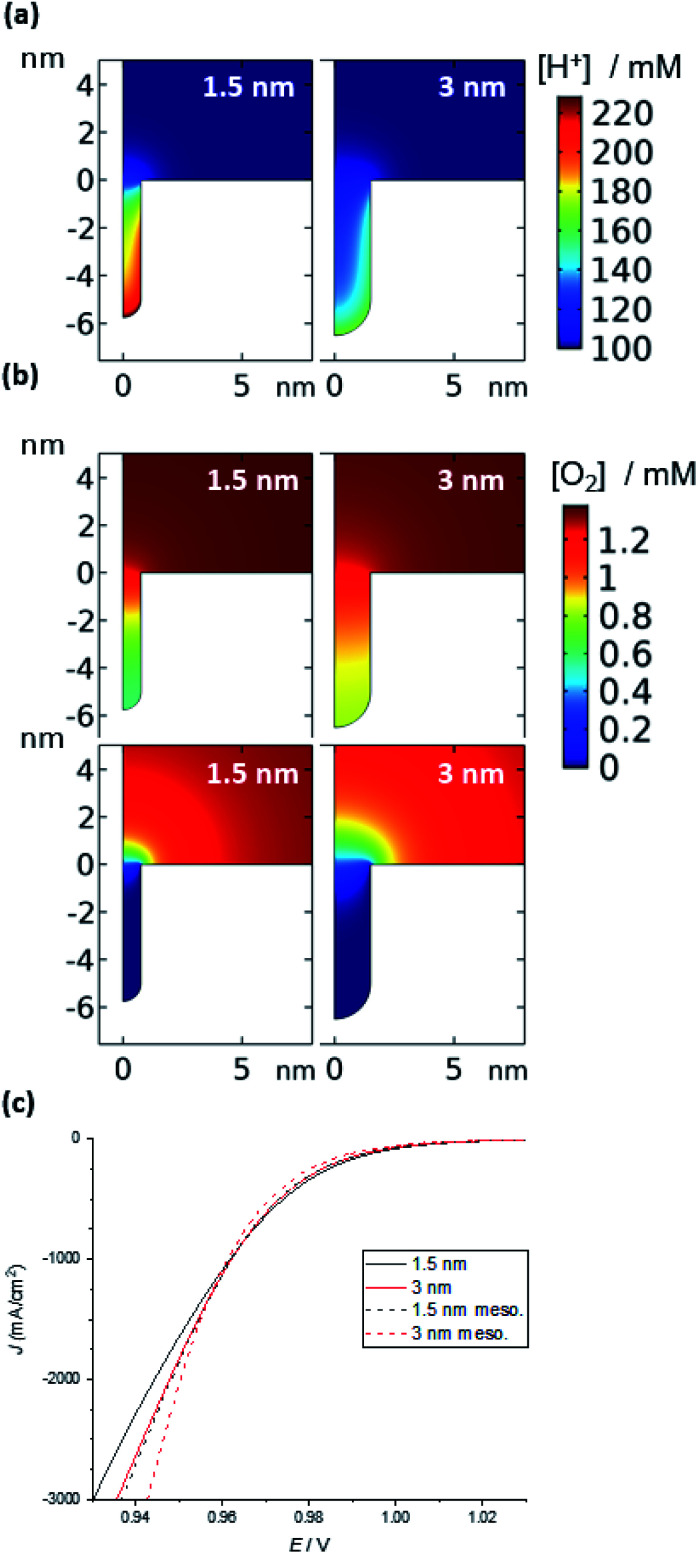
Simulation of the ORR in a nanozyme channel of varying diameters (1.5 and 3 nm). (a) Concentration of H^+^ in the absence of a reaction (1.1 V); the concentration is largely unperturbed during the ORR (see ESI Fig. S7†). (b) O_2_ concentration under mixed kinetic-diffusion (top) and pure diffusion (bottom). See Fig. S8† for examples at intermediate potentials and other channel and pore diameters. (c) Current density *vs.* applied potential at moderate overpotentials (see Fig. S11† for curves over the entire potential range).

In the presence of the same surface charge on the walls of the substrate channel for different substrate channel diameters (Fig. 5a), the model predicts that the concentration of protons inside the channels is higher the smaller the channel diameter. In each case, the excess charge in solution inside the substrate channel, which comes from an increase in proton concentration and a decrease in ClO_4_^−^ concentration, is equal and opposite to the charge on the surface. While in the smaller channels there is less surface area, the volume decreases more quickly with the radius (∼*r*^2^*vs.* ∼*r*), leading to higher concentrations (ESI Fig. S8†). It is also important to note that the concentration of H^+^ inside the substrate channel is significantly higher than in bulk solutions. This suggests that migration of H^+^ into the substrate channel will be the dominant mode of mass transport as H^+^ cannot diffuse against a concentration gradient. In contrast, the concentration of uncharged O_2_ inside the substrate channel is the same as in bulk solution regardless of the substrate channel diameter or depth (ESI Fig. S9†). Also, as it is uncharged, diffusion will be the mode of mass transport for this species.

Once the reaction is initiated, protons and oxygen will be consumed. The model predicts that under kinetic control at low overpotentials, when there is no appreciable depletion in O_2_, the difference in the concentration of protons inside the substrate channel results in a difference in reaction rates. Hence, it is suggested that the higher concentration of protons inside the channels of the nanozymes is responsible for the higher activity of the nanozymes compared to nanoparticles with the surfactant removed, with NZ_small_ having the highest specific activity. Note that, for the mesoporous nanoparticles, for the same width of pores as that of the substrate channel in the nanozyme, the model predicts a lower H^+^ concentration and hence a lower activity (Fig. S10†) as observed experimentally.

Under mixed kinetic-mass transport control, where the diffusion of oxygen into the substrate channel, distinct from that to the electrode surface, also influences the reaction rate, the model shows that there is depletion of O_2_ inside the channels. This depletion is greater for the narrower channels and at the bottom of the channels (Fig. 5b – top). That is, the reaction is limited by O_2_ diffusion into the channel. Note that, although protons are consumed in the reaction, there is no visible change in the concentration profiles for protons shown in Fig. 5a (1.1 V, prior to the onset of the reaction) from when the potential is changed to 0.7 V where the reaction is mass transport limited (ESI Fig. S8†). This confirms that the mass transport limitation comes from the diffusion of O_2_. This qualitatively agrees with the electrocatalysis results presented in Fig. 4 that show that as the overpotential becomes greater, the specific activity of NZ_medium_ and NZ_large_ becomes comparable or even greater than that of NZ_small_.

This experimental activity “cross-over” with increasing overpotential is also observed with the current density calculated from simulation (Fig. 5c). Finally, under mass transport control (Fig. 5b – bottom), the O_2_ concentration inside the channel is completely depleted for all channel diameters and the electrochemical reaction occurs predominantly at the entrance of the channel. As such, the entrance of the channel essentially serves as a ring nanoelectrode. For nanozymes with larger substrate channels, the larger circumference of the ring electrode gives a larger current and hence higher activity (note that of course the ESCA measurements determine the entire electrochemically active surface area, not the area available for the reaction in these non-uniformly accessible electrodes defined by the substrate channels).

We see similar results with the mesoporous particles (Fig. S10 and S12†). The model predicts that the mesoporous particles are less active in the kinetic regime than the nanozymes because the concentration of H^+^ inside the pore space is not as high as it is inside the substrate channels of the nanozymes. At the mass transport limitation, the mesoporous particles exhibit higher activity than the nanozymes for the same pore diameter. Again, this is because with the mass transfer limitation of O_2_ into the nanopores, the reaction occurs only at the pore entrance. Because the mesoporous particles have more pores, they have a larger surface area of pore entrances, higher ORR current and hence higher specific activity.

From these modelling results one explanation for the observed electrocatalytic responses as a function of applied potential can be derived. The model suggests that when electron transfer kinetics dominate, the concentration of H^+^ influences the reaction rate and smaller substrate channels with a higher concentration of H^+^ show higher specific activity. However, once mass transport starts to become important, the diffusive transport of oxygen into the substrate channel becomes a determining factor. Due to the non-uniformly accessible nature of the substrate channel, the reaction occurs mostly near the substrate channel entrance, and larger substrate channels present a larger accessible electrode area and hence a higher electrocatalytic current.

The modelling results also provide an explanation for why the etched nanoparticles with the surfactant removed show (1) lower specific activity than the nanozymes, (2) the specific activity increase with increasing substrate channel size at high overpotentials and (3) the attainment of a plateau with increasing overpotential. The explanation goes as follows: the oxygen reduction reaction at these nanoparticles occurs both on the external surface, which is not accessible with the nanozymes, and inside the substrate channels/mesopores. The response of the nanoparticles is influenced by the surface area of the nanoparticles that O_2_ can access before it becomes depleted. With high overpotentials many of the internal regions of the surfactant removed nanoparticles are not accessible to O_2_ but are included in the surface area determination. The higher activity of the nanozymes can be attributed to the very high concentration of H^+^ inside the substrate channel adjacent to the location of the ORR. In contrast, once the surfactant is removed, the ORR mostly occurs on the outer surface of the particle where the concentration of H^+^ is similar to that of the bulk, much lower than inside the nanochannels, and hence the specific activity is lower. The increase in activity for the surfactant removed nanoparticles with increasing substrate channel size is attributed to the greater flux of oxygen into the interior of the particle with larger channels and hence the higher activity. The origin of the plateau region is hypothesized to be related to the fact that mass transport of both hydrogen and oxygen to the nanoparticles could become limiting with these particles. With the nanozymes, the absence of a plateau shows that neither mass transport to the nanoparticles nor the number of active sites become limiting.

Finally, it is prudent to recall that the model does not seek to fit the data but assess whether the experimental data are consistent with nano-confinement. The model suggests this is the case but one needs to be cautious. As mentioned above with different channel diameters it is possible that there are different active sites. To give more confidence that nano-confinement is dominating the differences in experimental observation as a function of the substrate channel distribution, ORR measurements were performed at different electrolyte concentrations: 0.01 mol L^−1^, 0.1 mol L^−1^ and 1 mol L^−1^ HClO_4_ (Fig. S13†). Note that the estimated thickness of the electrical double layer (EDL) at each of these HClO_4_ concentrations is 3 nm, 1 nm and 0.33 nm respectively. At 1 mol L^−1^ HClO_4_ the specific activity is very similar across the entire potential range for NZ_small_ and NZ_large_. The similarity in activity suggests that the active sites in NZ_small_ and NZ_large_ are similar. At 0.1 mol L^−1^, the EDL thickness is 1 nm, causing EDL overlap on most of the channels of NZ_small_ with a concomitant higher activity than with NZ_large_ where most of the substrate channels will not have an EDL overlap. The higher activity when there is an EDL overlap is attributed to H^+^ being transported into the substrate channels *via* migration. Decreasing the concentration of the electrolyte further to 0.01 mol L^−1^ (EDL thickness = 3 nm) results in increased activity for both NZ_small_ and NZ_large_, as the EDL is thick enough to result in EDL overlapping on most of the channels even with NZ_large_. These results suggest that the differences in activity for the nanozymes with different channel sizes are predominantly due to a confinement effect although some contribution due to differences in active sites cannot be excluded.

## Conclusions

The impact of the diameter of the substrate channel in nanozymes was explored. The results show two key regimes where the diameter of the substrate channel has a very different impact on the electrocatalytic performance of the nanoparticles. At potentials where electron transfer kinetics dominate, it is suggested that the electrocatalytic reaction occurs along the entire substrate channel and the greater the nano-confinement (the smaller the substrate channel diameter) the higher the electrocatalytic performance. However, at higher overpotentials, where the reaction is mass-transport limited, physicochemical modelling supports the hypothesis that the electrocatalytic reaction occurs predominantly at the entrance of the substrate channel. In this regime the larger the substrate channel, the higher is the specific activity of the electrocatalyst. As such, in the mass transport regime nano-confinement does not have an impact on the electrochemical reaction.

These results indicate how nano-confinement can have a profound impact on electrocatalytic activity by concentrating the required reactants. In the case of the ORR used as a model to demonstrate the concept, modelling suggests that the need for charge compensation of surface potential on the walls of the substrate channel sees protons as counter ions pre-concentrated inside the substrate channel *via* migration. As protons are also the reactant, the higher concentration of the reactant results in a higher specific activity. This migration driven concentration of the reactants into the substrate channel, to improve the reaction rate, can be thought of as analogous to active transport of reactants in enzymes, thus extending the mimicry of the enzyme mechanism with our nanozyme particles. The nano-confinement facilitating the reaction rate is just one way in which the nano-confinement may enhance electrocatalytic reactions. If a bimolecular reaction is involved, then the nano-confinement can also increase the rate by more closely locating the two reactants and give a different product distribution, as we showed recently with nanozymes for the carbon dioxide reduction reaction.^14^

## Methods

### Nanozyme synthesis

The nanozyme particles were prepared by acid etching of carbon supported Pt–Ni nanoparticles stabilized with oleylamine. Pt–Ni nanoparticles of three stoichiometric ratios were obtained with a synthesis method adapted from the literature (Pt–Ni1.5, Pt–Ni2.5, and Pt–Ni3.0).^12^ Briefly, to synthesise Pt–Ni3.0 nanoparticles, a 3 : 1 molar ratio of Ni(acac)_2_ and Pt(acac)_2_ was dissolved in oleylamine at 100 °C, de-gassed and reacted for 1 h at 300 °C under argon. The resulting nanoparticles were then washed with ethanol to remove excess surfactant. The nanoparticles were further supported on carbon Vulcan (XC 72R Cabot) by mixing hexane-dispersed nanoparticles with carbon powder under sonication for 2 h. The hexane was then removed, and the carbon supported nanoparticles were washed with ethanol and dried at room temperature. For etching, the supported nanoparticles were dispersed in HNO_3_ 70% and kept under sonication for 1 min, followed by centrifugation, washing 3 times with water and 2 times with ethanol to remove excess of acid, Ni^2+^ and surfactant and drying at room temperature. The etching procedure was repeated twice to create mesoporous nanoparticles. Non-etched nanoparticles without the surfactant and etched nanoparticles without the surfactant were used as controls. The surfactant was removed by exposure to a 200 °C air flow for 5 h inside a tube furnace.

### Inductively coupled plasma-optical emission spectrometry (ICP-OES)

First, the carbon supported particles were digested in aqua regia for 1 h at 80 °C to dissolve the metals, diluted with water and then analysed using an Optima7300DV-ICP-OES PerkinElmer instrument at the selected wavelengths of Ni 231.604 and Pt 265.945 to give the molar composition of the nanoparticles.

### Scanning-transmission electron microscopy (S-TEM)

TEM and STEM imaging were performed on a JEOL JEM-F200 (200 kV, cold field emission gun) equipped with an annular dark-field (ADF) detector and a JEOL windowless 100 mm^2^ silicon drift X-ray detector. STEM images were acquired with a convergence semi-angle of 8.2 mrad or 62 mrad (for achieving high-angle *Z*-contrast conditions). TEM specimens were prepared by drop casting of a dispersion of nanozymes without a carbon support in hexane on carbon coated copper grids, rinsing with warm ethanol and allowing to dry under ambient conditions.

### Electrochemical characterisation

#### Working electrode preparation

2.5 mg of the carbon supported nanoparticles were dispersed in 750 μL of H_2_O, 249 μL of isopropyl alcohol and 1 μL of Nafion 5% solution to give an ink. 6 μL of the ink was placed on an RDE glassy carbon disk (0.07 cm^2^) and dried at 120 °C for 1 min to give a thin and uniform film.

#### Electrochemical setup

All experiments were performed using a μAutolab potentiostat controlled with Nova 2.1.2 software using an electrochemical cell with a three-electrode assembly consisting of a Pt mesh and Ag|AgCl|3 mol L^−1^ KCl as the counter and reference electrodes, respectively. The reference electrode was separated from the main cell compartment using a fritted double-junction filled with the electrolyte to avoid chloride contamination. Three different electrolytes were used: 1 mol L^−1^ HClO_4_, 0.1 mol L^−1^ HClO_4_, and 0.01 mol L^−1^ HClO_4_ (Suprapur – Merck). The cell was kept under N_2_ or O_2_ by purging during the experiments. All potentials are referenced against the reversible hydrogen electrode (RHE) and were converted by measuring the potential difference in the electrolyte between the reference electrode used for the measurements and a fresh RHE prepared prior to the experiments.

#### Electrochemical activation

Catalyst activation was achieved by cycling the potential under N_2_ from 0.04 to 1.00 V (nanozymes and etched particles) and 0.04 to 1.40 V (non-etched particles) at a scan rate of 200 mV s^−1^ until no differences in the voltammograms were observed (typically 10–20 cycles).

#### Electrochemically active surface area (ECSA)

Two different methods were used: (a) integration of the reduction current in the H_UPD_ region (after excluding the capacitive current) of the last voltammogram cycle during activation to give the charge which was further converted into the surface area (using 210 μC cm^−2^ as correlation). (b) Electrodeposition of a Cu monolayer at 0.49 V (RHE) for 3 min using 5 mM CuSO_4_ in 0.1 mol L^−1^ HClO_4_ as the electrolyte followed by its oxidation by linear sweep voltammetry at 100 mV s^−1^ up to a potential of 1.00 V. The background subtracted oxidation currents were then integrated to give the charge which was further converted into the surface area (using 420 μC cm^−2^ as correlation). Further details are given in Section S2 of the ESI.†

#### Oxygen reduction reaction electrocatalysis

The measurements were done by linear sweep voltammetry in the positive direction from 0.04 to 1.00 V at 100 mV s^−1^. The electrolyte was saturated with O_2_ and the electrode was rotated at 400–3200 rpm (at 400 rpm increments) using an Autolab RDE2. Background measurements were done under the same conditions under saturated N_2_, and the current was subtracted from the currents measured under O_2_. The potentials were corrected for *iR* drop by measuring the solution resistance at OCP by electrochemical impedance spectroscopy.

#### Experimental order

Background measurements were performed after experimental measurements for the nanozyme samples to avoid surfactant removal due to electrochemical oxidation of the platinum surface.

#### Finite element modelling

A numerical model that solved the Poisson–Nernst–Planck equations for the concentrations of O_2_, H^+^, and ClO_4_^−^ and the electric potential, and which described the electron transfer by a 4-electron Tafel process, was implemented in a commercial finite element package, Comsol Multiphysics v5.4. A detailed description of the model is provided in the ESI, Section S4,† with additional details available in the automatically generated Comsol ‘model report’, which is provided as a separate ESI file.†

## Conflicts of interest

There are no conflicts to declare.

## Supplementary Material

SC-011-C9SC05611D-s001
