# Supplementary material for: The importance of nanoscale confinement to electrocatalytic performance
Source: Chem Sci. 2019 Dec 11;11(5):1233–40. doi: 10.1039/c9sc05611d (PMC8148078; doi:10.1039/c9sc05611d)
Supplement: SC-011-C9SC05611D-s001 [file SC-011-C9SC05611D-s001.pdf]

Electronic Supplementary Material (ESI) for Chemical Science.
This journal is © The Royal Society of Chemistry 2019

Supplementary Information

The importance of nanoscale confinement on electrocatalytic performance

J. Wordsworth, T. M. Benedetti, A. Alinezhad, R. D. Tilley, M. A. Edwards, W. Schuhmann, J. J. Gooding

Contents

S1: HAADF-STEM images of etched Pt-Ni nanoparticles......2

S2: Calculation of particle areas from electrochemical underpotential deposition......3

S3: TEM images before and after the electrochemical measurements......4

S4: Voltammograms for NZ_{small} and NZ_{medium}......5

S5: Koutecký-Levich analysis of ORR kinetics......6

S6: Description of finite element simulations......7

S7: Simulated H^+ and O_2 concentrations in nanozymes and mesoporous nanoparticles and simulated current densities in nanozymes......10

S8: References......15

S1: HAADF-STEM images of etched Pt-Ni nanoparticles

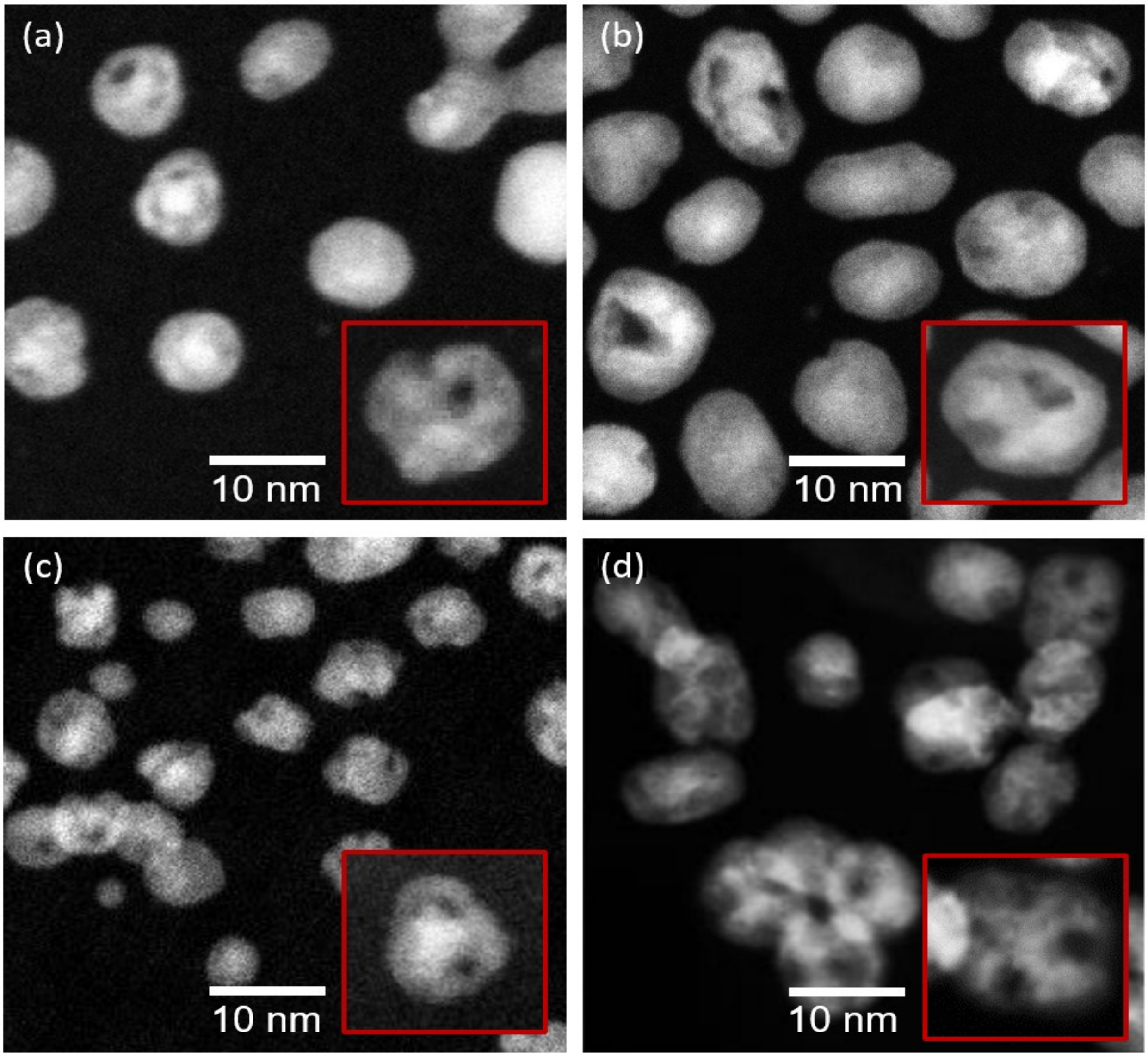

Figure S1. HAADF-STEM images of etched Pt-Ni nanoparticles (a) NZ_{small}, (b) NZ_{medium}, (c) NZ_{large}, and (d) mesoporous nanoparticles. Image insets not to scale. These provide further examples to supplement the images shown in Figure 1 of the main text.

S2: Calculation of particle areas from electrochemical underpotential deposition

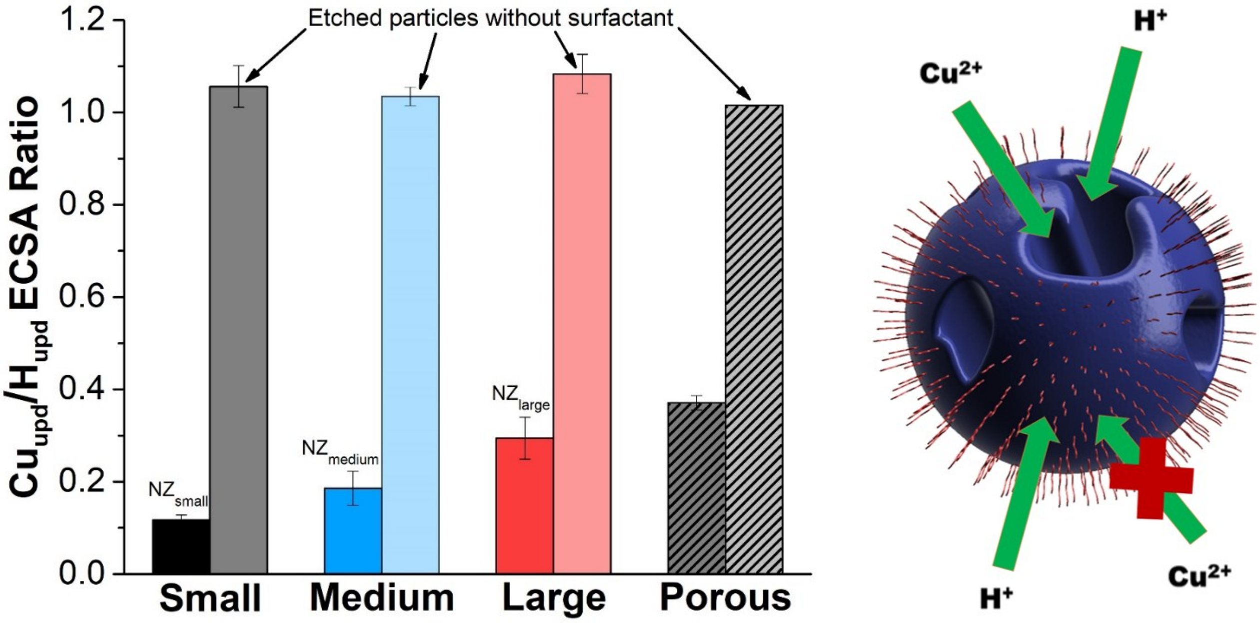

Figure S2. The ratio between the ECSA calculated by Cu_{UPD} and H_{UPD} gives information about the contribution of the channels to the total surface area of the nanoparticles. This is due to the ability of H^+ to partially permeate through the surfactant layer, also forming a monolayer on the passivated Pt surface. Cu^{2+} ions, being larger than protons, are only able to electrodeposit on the non-passivated Pt surface inside the channels.

For particles without surfactant, this ratio is greater than unity. As surfactant is no longer hindering the Cu^{2+} ions ability to adsorb to Pt surface, both ECSA methods are measuring the same surface area. Ratio values greater than one occur from the underestimation of ECSA from H_{UPD} due to strain effects induced by subsurface Ni atoms.[1]

It can be seen from Figure S2 that this ratio increases for the nanozymes as initial Ni content increases.

S3: TEM images before and after electrochemical measurements

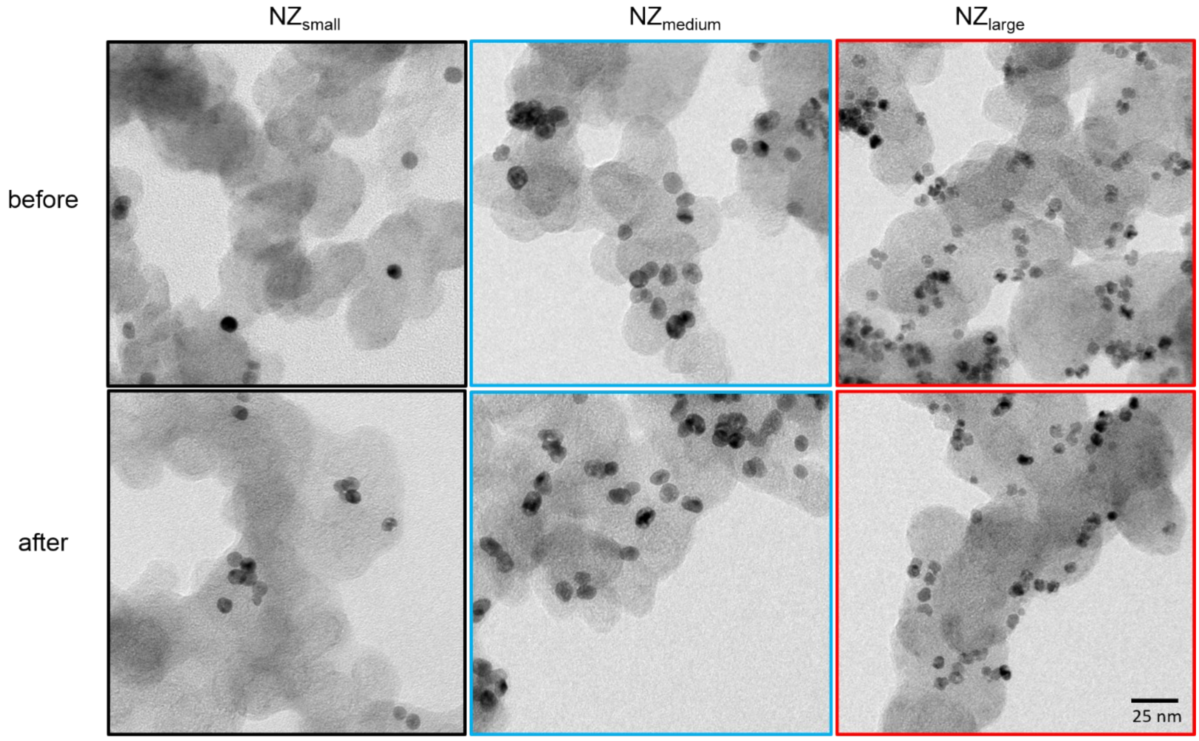

Figure S3: low magnification TEM of nanozymes supported in carbon before (top) and after (bottom) the electrochemical measurements. NZ_{small} (black), NZ_{medium} (blue) and NZ_{large} (red)

S4: Voltammograms for NZ_{small} and NZ_{medium}

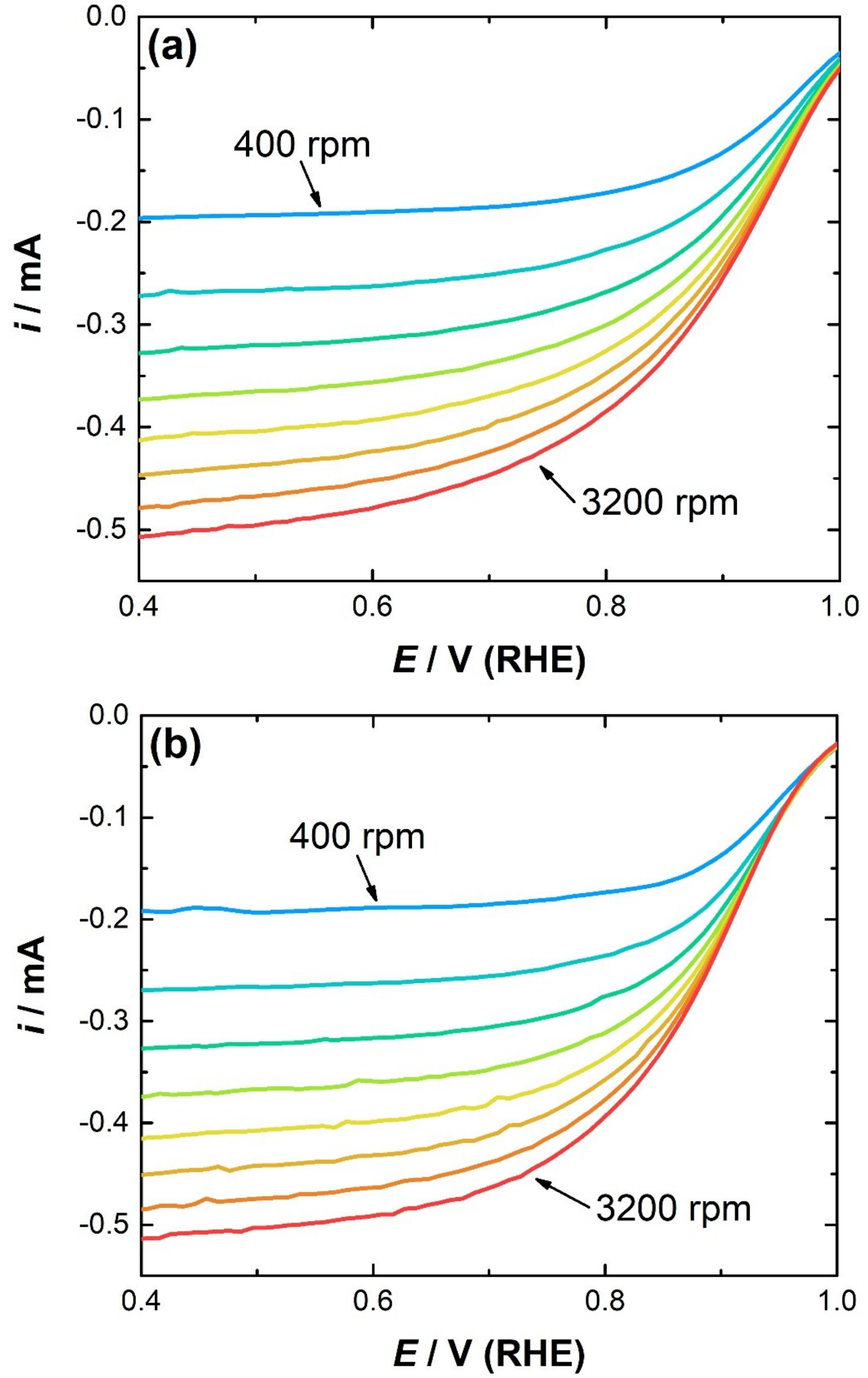

Figure S4. Background subtracted and iR corrected LSVs of (a) NZ_{small} and (b) NZ_{medium} at rotation speeds from 400–3200 rpm (400 rpm steps) in O_2 saturated electrolyte. The scans were performed in 0.1 mol L^{-1} $HClO_4$ in the anodic direction at a scan rate of 100 mV s^{-1}.

S5: Koutecký-Levich analysis of ORR kinetics

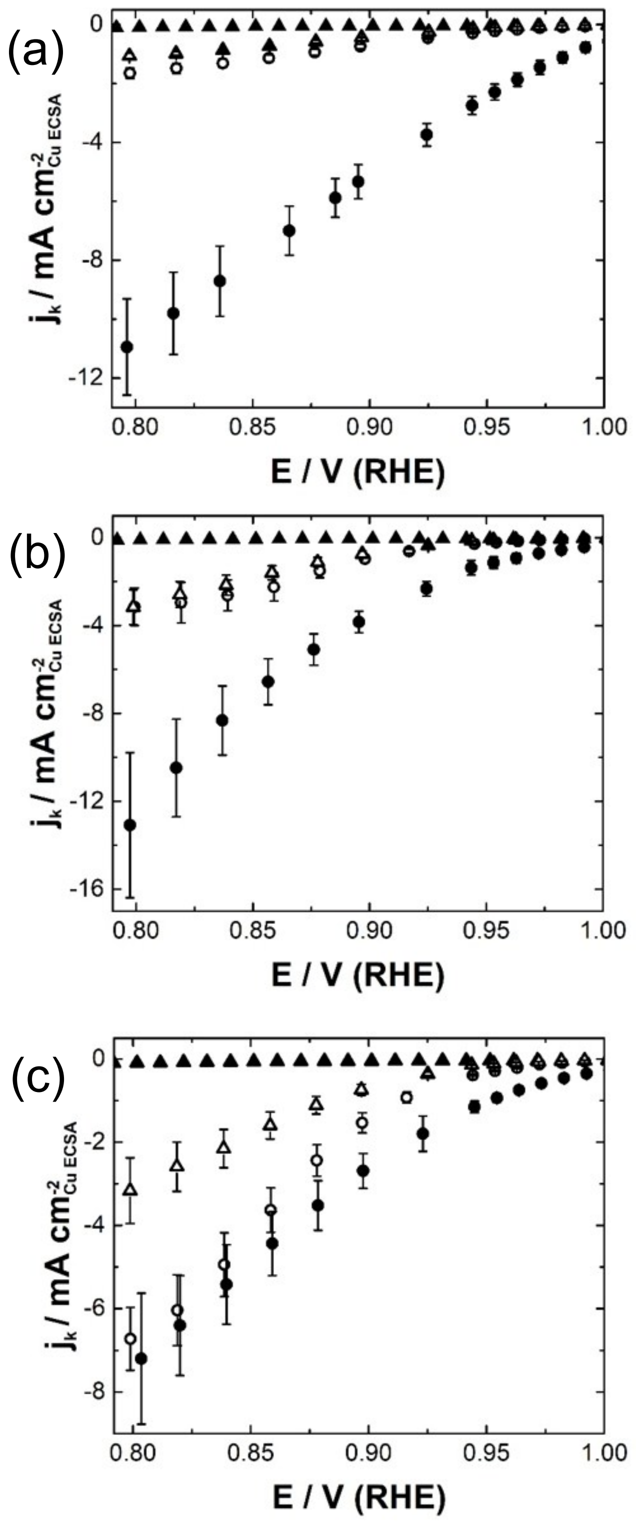

Figure S5. Average kinetic currents calculated using Koutecký-Levich plots from 0.8 – 1.0 V (RHE) for surfactant covered

particles (closed shapes), particles with surfactant removed (open shapes), etched particles (circles), and unetched particles (triangles), for (a) NZ_{small}, (b) NZ_{medium}, (c) mesoporous particles.

S6: Description of finite element simulations

A model was developed to understand the activity response of the nanozymes and mesoporous particles as a function of their geometry. The distributions of concentrations of solution species, c_i (i = H^+, O_2, ClO_4^-), the electric potential, ϕ, and the current response of a single pore within a nanozyme, were calculated through solution of the coupled Poisson-Nernst Planck equations:

$$J_i = - D_i \nabla c_i - \frac{z_i F}{RT} D_i c_i \nabla \phi \tag{S1}$$

$$\nabla^2 \phi = - \frac{F}{\epsilon_0 \epsilon_r} \sum_i z_i c_i \tag{S2}$$

where J_i, D_i and z_i are, respectively, the flux, diffusion coefficient and charge of species i; with the values of D_{ClO4-} = 1.79 × 10^{-5} cm^2/s,[2] D_{H+} = 7.1×10^{-5} cm^2/s,[2] D_{O2} = 1.67 × 10^{-5} cm^2/s.[3] The relative permittivity of the aqueous solution was set at ϵ_r = 78. For simplicity, this description ignores finite ion size effects.

For understanding the nanozyme response, Equations S1 and S2 were solved in the idealized axisymmetric geometry shown in Figure S5 a/b, which represents a single pore. As with other assumptions made in the simulation (*vide infra*), the hemisphere-capped cylinder geometry was chosen to capture the important properties of a nanozyme pore (depth, width), while not attempting to describe the precise variations observed in the experimental system.

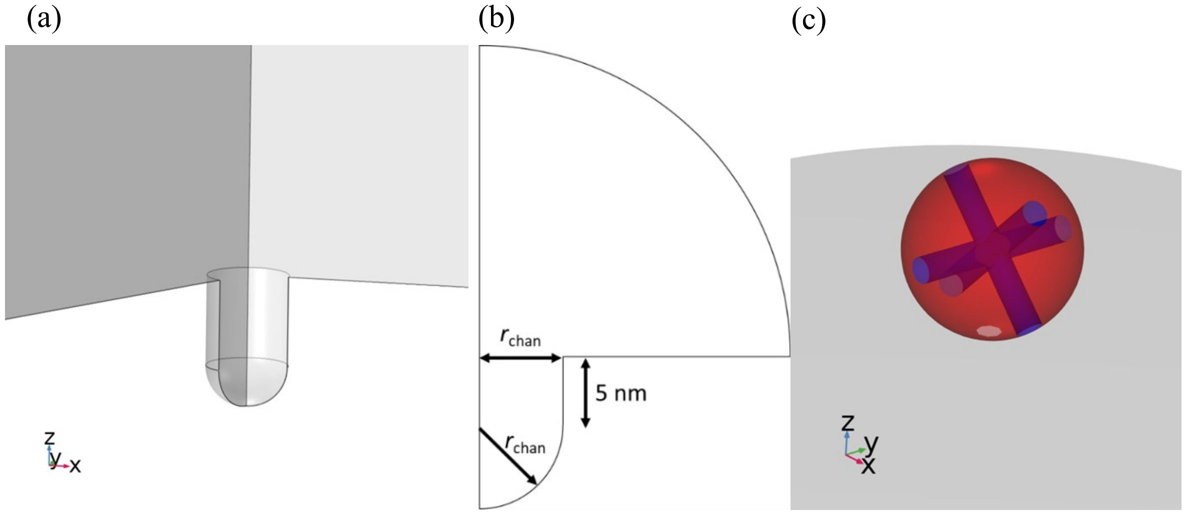

Figure S6. (a) and (b) Geometry of a single channel approximated as a hemispherically-capped cylindrical channel. (a) full 3D geometry; (b) 2D axisymmetric, as used for simulations (not to scale). r_{chan} is the channel radius. (c) Geometry of mesoporous particle (red) with 3 orthogonal channels (blue) sitting on a planar surface (grey).

The surface beyond the pore represents the passivated surface of the particle. The lack of activity is described by a 'no flux' boundary condition for all of the solution species

$$J_i \cdot \underline{n} = 0 \tag{S3}$$

where $\underline{n}$ is the inward pointing unit normal to the surface. In the results presented in this work, we make the assumption that this surface displays no charge, while noting that the behavior observed is qualitatively insensitive to the any charge here.

$$\nabla\phi \cdot \underline{n} = 0 \tag{S4}$$

On the curved boundary far from the pore (Figure S6b), representing bulk solution, we match the experimental bulk concentration (c_{H+}=c_{ClO4-} = 0.1 M and c_{O2} = 1.38 mM) and define a reference potential of 0 V.

The axis of symmetry possesses no charge and no flux of any species can occur across it (Eqs. S3 and S4). The planar surface upon which the mesoporous particle sits (grey in Figure S5c) is also uncharged and impermeable.

The potential and ion distribution proximal to a metallic surface, the electrical double layer, is a function of the potential applied to the metal, the chemical nature of the surface and the species in solution (*cf.* specifically adsorbed ions). Numerous works have been devoted to the study of the electrical double layer.[4] We make no attempt to capture the full character of this response, which would include multiple parameters for which there is scarce information (energies of specific ion adsorption, potential of zero charge), all of which are a function of the potential of the metal and include influence from its history (e.g., surface oxides). Instead, we aim to qualitatively capture this influence in a single parameter, the surface charge, which we set to a constant value (-5 mC/m^2). This allow qualitative comparison between different pore geometries while minimizing the number of free parameters.

$$\epsilon_0\epsilon_r\nabla\phi \cdot \underline{n} = 5\ mC/m^2 \tag{S5}$$

Electron transfer on the Pt surface was described by a Tafel kinetic expression.

$$J_{O_2} \cdot \underline{n} = 4J_{H^+} \cdot \underline{n} = -kc_{O_2}\frac{c_{H^+}}{c_{H^+,init}}\exp\left(-\frac{4\alpha F}{RT}\eta\right) \tag{S6}$$

Due to the large overpotential, η, it is reasonable to consider only the forward reaction. We assume all reactions proceed through the overall reaction (→ H_2O), i.e., no H_2O_2 is produced. We take the rate constant, $k = 1\times10^{-5}$ cm/s and $\alpha = 0.5$, which give an onset potential in a similar range to observed in the experiments. However, as we are making qualitative comparisons between different nanozyme geometries using a fixed description of ET, no effort was made to precisely fit the experimental data and the precise choice of these parameters is unimportant and has no influence on the trends reported. The concentrations in equation S6 are the spatially varying concentrations on the Pt surface. Electron tunneling was ignored and electron transfer assumed to take place only on the unpassivated surface of the channel/pore.

The current (density) was calculated from the integral of the normal flux of O_2 over the surface of the pore. As expressed in

$$\text{Current} = 4\int_{pore\ surface} \underline{n} \cdot J_{O_2} \tag{S7}$$

where the integral represents either a surface integral or an integral of rotation for the mesoporous particle or the nanozyme, respectively.

The equations were discretized over a mesh as shown in Figure S7 using the commercial finite element modelling package Comsol Multiphysics (version 5.4). Accuracy of simulations was confirmed by invariance of the solution on a finer mesh, with tighter numerical tolerances and

a larger simulation domain. To aid in replication of the simulations performed in this work, an automatically generated Comsol 'model report' has been uploaded as a separate supporting information file, with included addition information on the solver settings, the formulation of the mesh, etc.

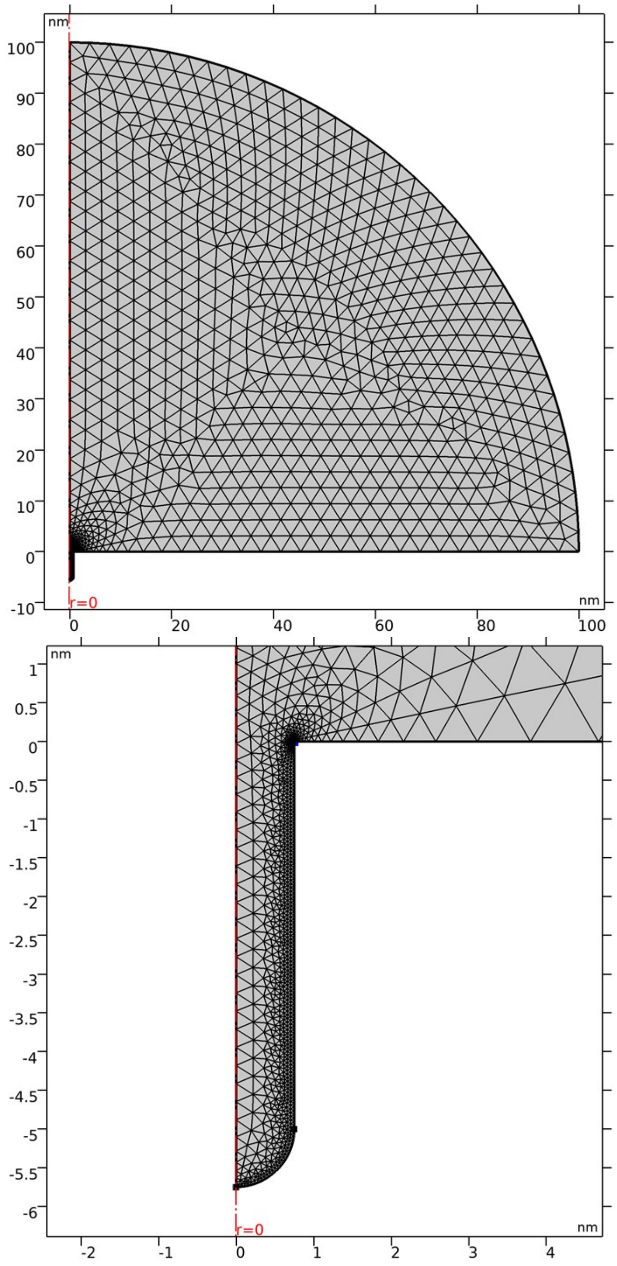

Figure S7. Representative mesh for the full simulation domain (left) and zoomed-in to show the channel (right) upon which equations S1 and S2 were discretized and solved. The mesh on the surface of the Pt was set to a size not greater than 1/20th of the Debye length to ensure the concentration and electric potential in this region were accurately captured.

S7: Simulated H^+ and O_2 concentrations in nanozymes and mesoporous nanoparticles and simulated current densities in nanozymes

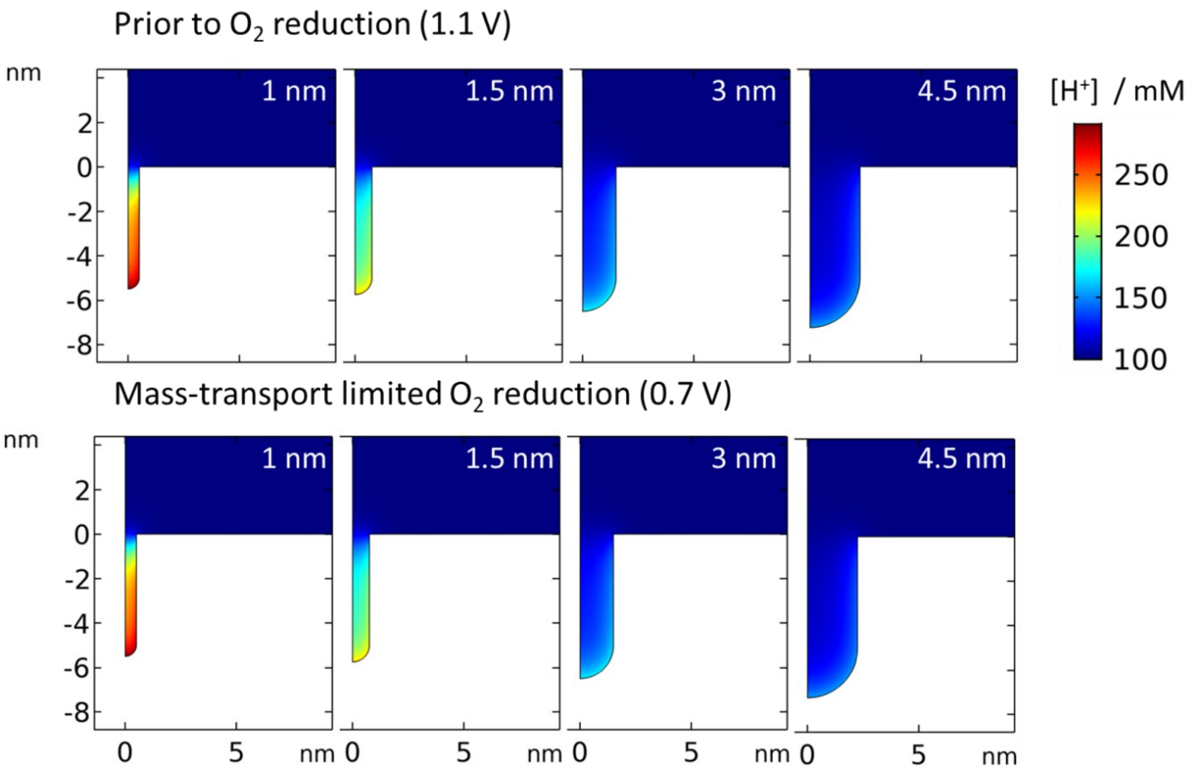

Figure S8. Simulated H^+ concentration distribution in nanozyme channels of different diameters (labelled) prior to ORR onset (top, 1.1 V) and at diffusion limited reduction of oxygen (bottom, 0.7 V) show that depletion of H^+ is insignificant.

Figure S8 shows the proton concentration in a nanozyme channel both before the onset of ORR (1.1 V, top) and when the reaction occurs at a diffusion limited rate (0.7 V. bottom). For each channel size, the proton concentration in each situation is essentially indistinguishable (minor (<~1 mM) changes are calculated). This justifies our decision to present proton concentration profiles taken at a single potential as a proxy for those concentrations at all rates (Figure 5a, main text).

This result is unsurprising when one considers that the saturated O_2 concentration of 1.38 mM is considerably lower than the 100 mM bulk concentration of H^+. Even though four protons are consumed for each O_2 reduced, the higher diffusivity of the protons limit the change to a concentration comparable to the bulk O_2 concentration.

In our simple model of a mesoporous particle, the predicted H^+ concentration is as shown in Figure S8. As with the simulated nanozymes, in the model this concentration does not change with the rate or ORR (not shown) and shows higher proton concentrations with narrower diameters.

1.0 nm diameter

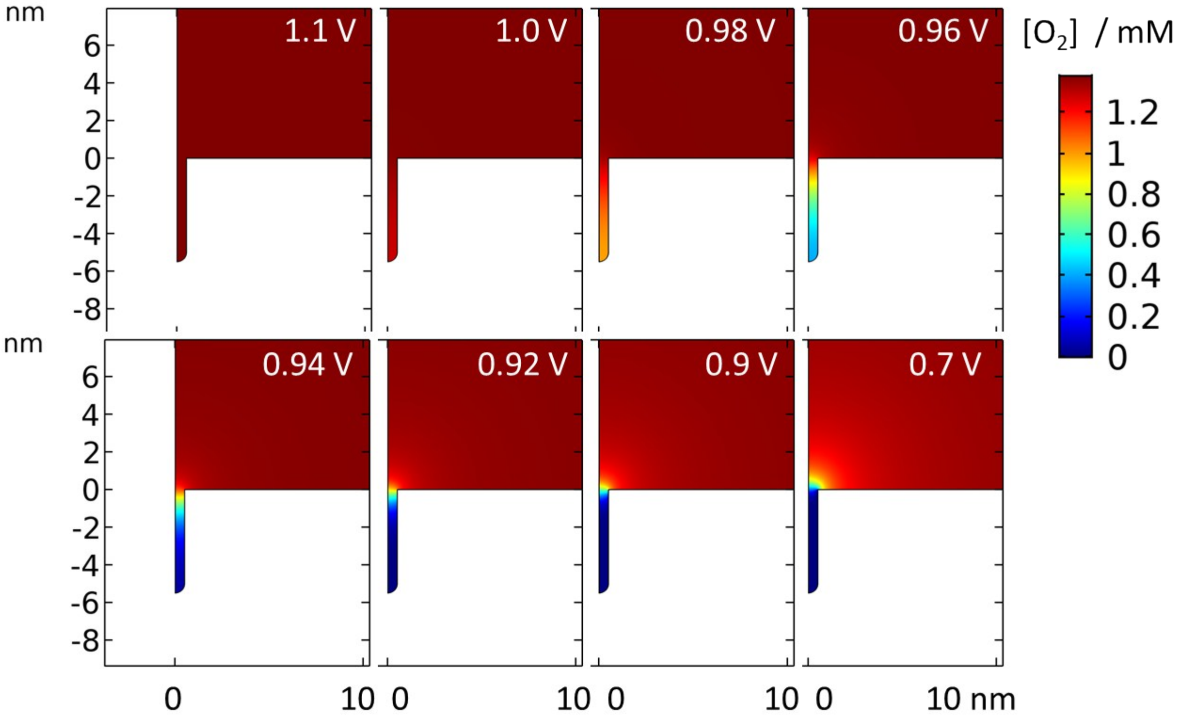

1.5 nm diameter

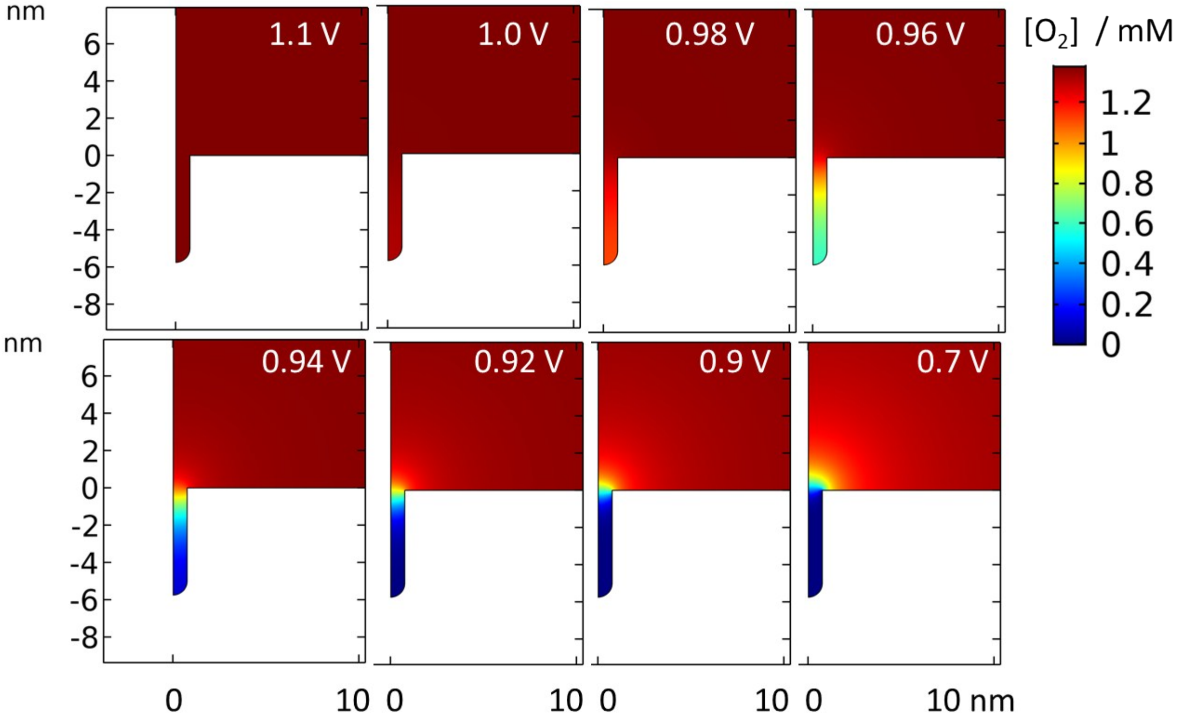

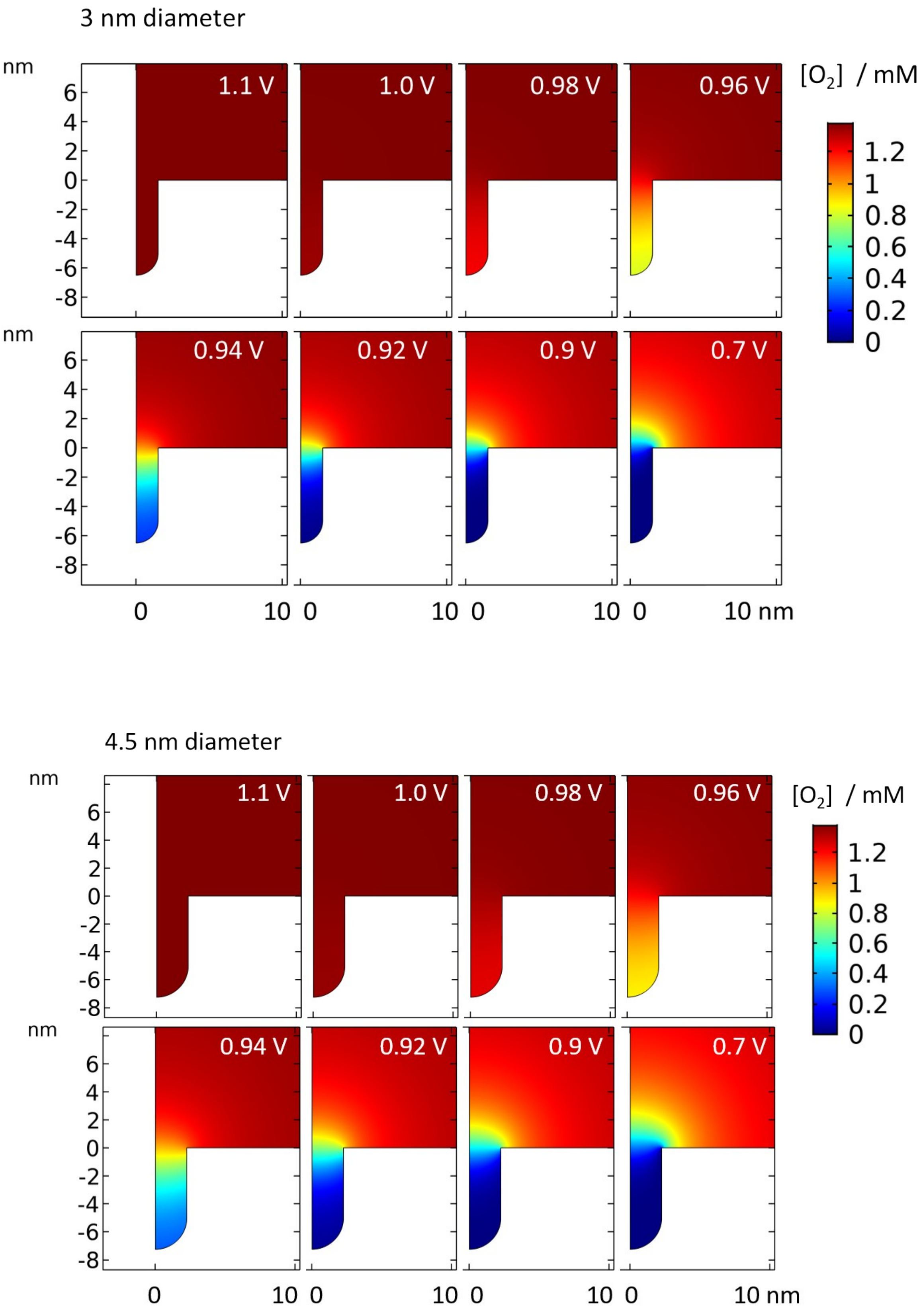

Figure S9. Simulated O_2 concentrations during ORR as a function of potentials for a range of channel sizes.

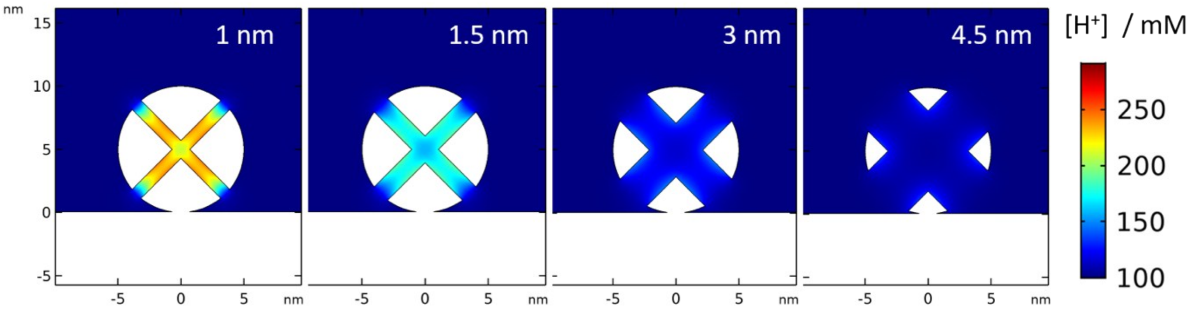

Figure S10. Simulated H$^+$ concentration with a 5 nm radius mesoporous particle for a range of pore diameters (as labelled) prior to ORR onset (1.1 V). H$^+$ depletion is insignificant as overpotential is increased, as previously shown for nanozymes in Figure S7. Cross-section cuts through the center of the particle, a third channel runs in the dimension perpendicular to the page (see geometry in Figures S5c).

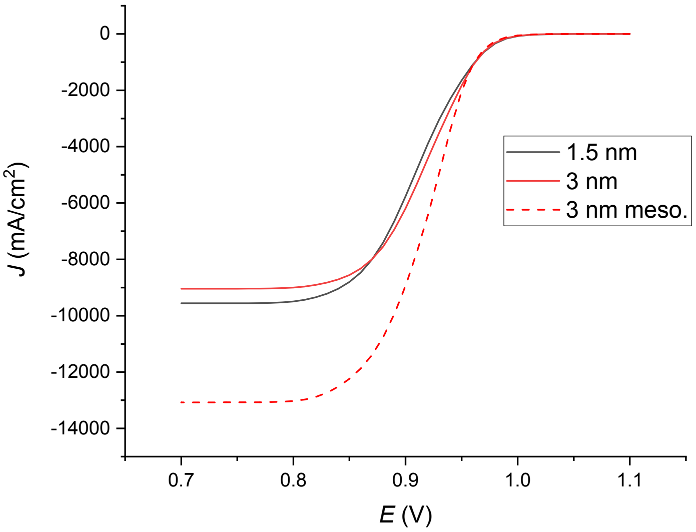

Figure S11. Simulated current densities in nanozymes over a wide potential range. This plot replots data that were shown in Figure 5 in the main text over a smaller potential range. At higher potentials, the current reaches a transport limited plateau.

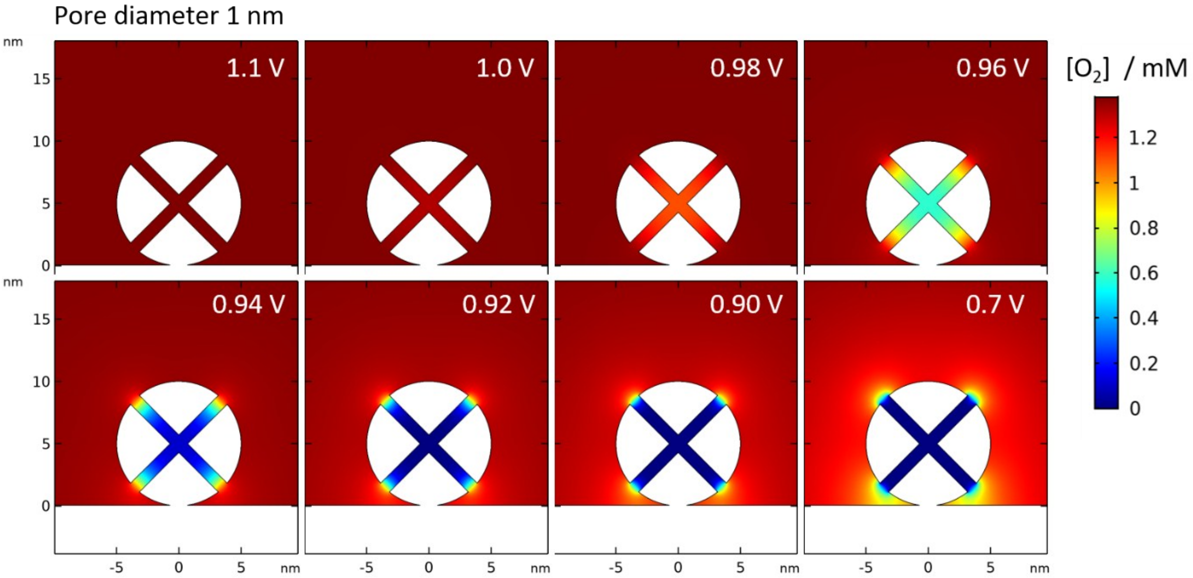

Pore diameter 1 nm
1.1 V
1.0 V
0.98 V
0.96 V
0.94 V
0.92 V
0.90 V
0.7 V
[O2] / mM
1.2
1
0.8
0.6
0.4
0.2
0
nm
15
10
5
0
-5
0
5

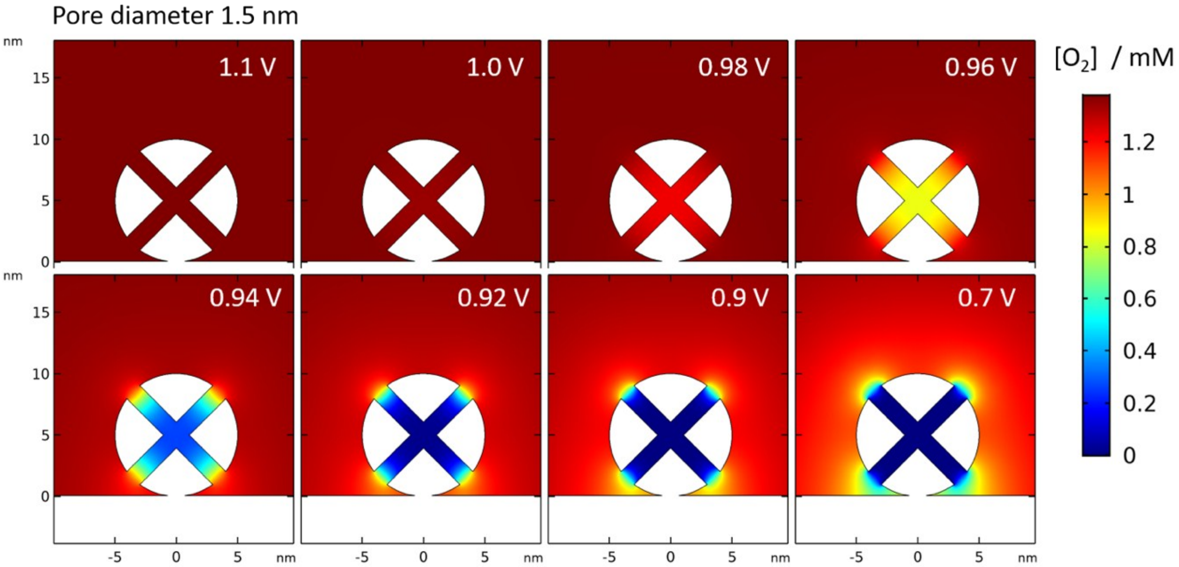

Pore diameter 1.5 nm
1.1 V
1.0 V
0.98 V
0.96 V
0.94 V
0.92 V
0.9 V
0.7 V
[O2] / mM
1.2
1
0.8
0.6
0.4
0.2
0
nm
15
10
5
0
-5
0
5

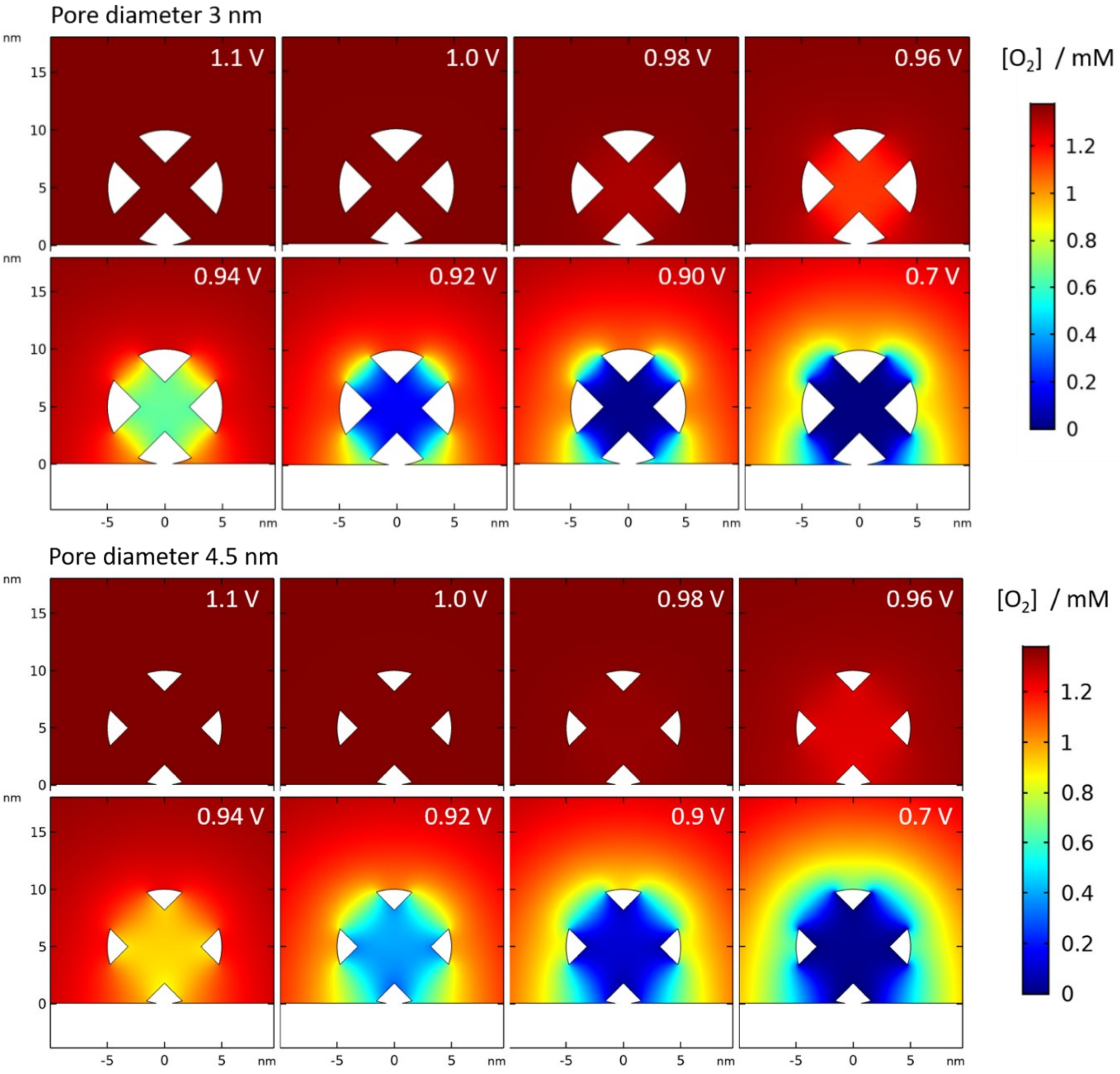

Figure S12. Simulated O_2 concentration with a 5 nm radius mesoporous particle for a range of pore diameters and potentials (as labelled). Cross-section cuts through the center of the particle, a third channel runs in the dimension perpendicular to the page.

The plots in Figures S9 and S12 show simulated O_2 concentrations over a wider range of pore sizes and potentials, supporting those shown in Figure 5b of the main text and displaying identical trends.

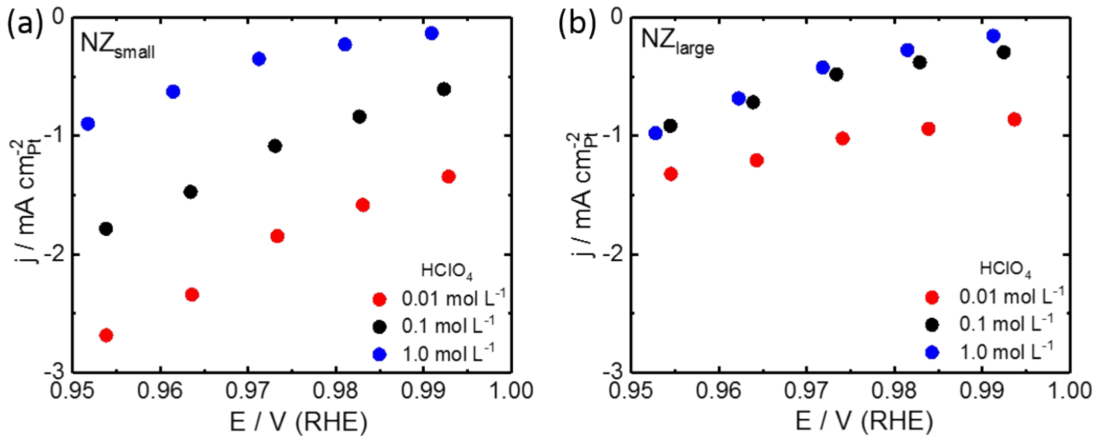

Figure S13: Kinetic current densities calculated using Koutecký-Levich plots from 0.95 – 1.0 V (RHE) for (a) NZ_{small} and (b) NZ_{large} at different $HClO_4$ concentrations: 0.01 mol L^{-1} (red), 0.1 mol L^{-1} (black) and 1.0 mol L^{-1} (blue).

S8: References

1. van der Vliet, D. F.; Wang, C.; Li, D.; Paulikas, A. P.; Greeley, J.; Rankin, R. B.; Strmcnik, D.; Tripkovic, D.; Markovic, N. M.; Stamenkovic, V. R. Unique electrochemical adsorption properties of Pt-skin surfaces. *Angew. Chem., Int. Ed.* **2012**, 51 (13), 3139−42.

2. "Ionic Conductivity and Diffusion at Infinite Dilution" in CRC Handbook of Chemistry and Physics, 100th Edition (Internet Version **2019**), John R. Rumble, ed., CRC Press/Taylor & Francis, Boca Raton, FL

3. Wakabayashi, N.; Takeichi, M.; Itagaki, M.; Uchida, H.; Watanabe, M. Temperature-dependence of oxygen reduction activity at a platinum electrode in an acidic electrolyte solution investigated with a channel flow double electrode. *J Electroanal Chem*. **2005**, 574, 339-346.

4. Bard, A. J.; Faulkner, L. R., *Electrochemical Methods: Fundamentals and Applications, 2nd Edition*. Wiley Textbooks: 2000.
